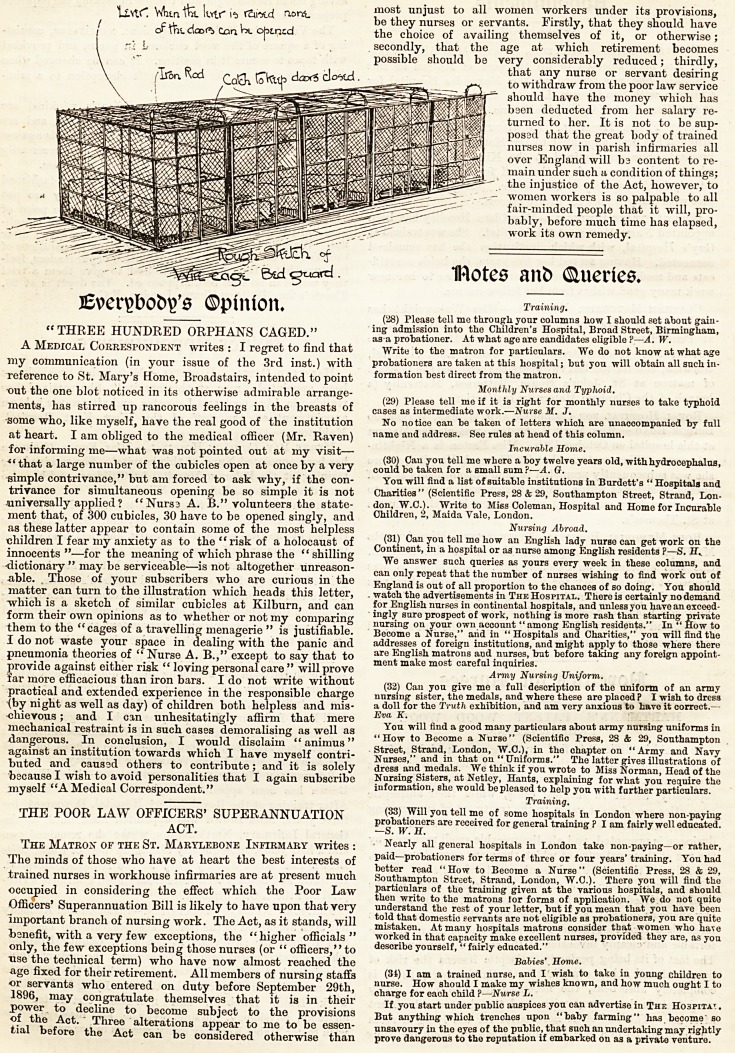# The Hospital Nursing Supplement

**Published:** 1896-10-24

**Authors:** 


"The Hospital" Nursing Mirror.

Being the Extra Nursing Supplement of "The Hospital."

[Contributions for this Supplement should be addressed to the Editor, The Hospital, 28 & 29, Southampton Street, Strand, London, W.C., and should have the word "Nursing" plainly written in left-hand top corner of the envelope.]

### News from the Nursing World.

### THE PRIMROSE LEAGUE AND THE Q.V.J.I.

At a meeting of the Grand Council of the Primrose League, held at the offices in Westminster on October 15th, it was resolved that the League should commemorate the sixtieth year of Her Majesty's reign by raising throughout its branches a substantial sum to aid the endowment of the Queen's Jubilee Institute for Nurses.

### A GIFT FOR QUEEN CHARLOTTE'S HOSPITAL.

The Duchess of York is a vice-patron of Queen Charlotte's Lying-in Hospital, and Her Royal Highness has just given acceptable proof of her interest in that ancient institution by presenting a donation of £10 to the extension and improvement fund.

### THE ARCHBISHOP OF CANTERBURY AND ST. THOMAS'S HOSPITAL.

The late Archbishop of Canterbury took a special interest in St.Thomas's Hospital. When he was its near neighbour in residence at Lambeth his chaplains visited the wards regularly, and the private garden at the Palace was placed by Archbishop Benson at the disposal of the hospital staff. Speaking of these things in his sermon in the hospital chapel on Sunday, the Rev. J. Grant Mills said the loss to the hospital was that of a personal friend.

### A NURSING HOME AT MAIDA VALE.

A Medical and Surgical Home has been started this summer by Miss Ellison, L.O.S., and member of the Society of Trained Masseuses, at 258, Elgin Avenue, Maida Vale. It is in a delightfully airy, open, and convenient part of London ; the house is very well adapted to its purpose, and comfortably and suitably furnished. Miss Ellison has another trained nurse to help her, and especially undertakes cases requiring rest and massage, and Weir-Mitchell or Nauheim treatment.

### OVERWORKED NURSES.

It is to be hoped that a correspondent of the *Leicester Daily Post*, who wrote recently concerning the hours of work of the nurses at the Borough Fever Hospital, has been misinformed, or the remark that they constitute "a black mark on the fair fame of Leicester" is quite too mild an expression of opinion. "A day nurse," it is said, "is expected to be on duty a little after six o'clock in the morning and continues so until eight o'clock in the evening, with just a brief interval for meals, and this . . . . seven days per week." It cannot be possible that the Sanitary Committee of Leicester allows the nurses in their employ no off-duty time before eight o'clock in the evening seven days in the week. We should be glad to know what are the actual facts of the case.

### TRAINED NURSES' CLUB.

The sale of work annually held at the Midwives' Institute and Trained Nurses' Club (12, Buckingham Street, Strand) will take place this year on December 3rd and 4th, and between now and then Mrs. Nichol will gladly welcome any contributions towards it. All kinds of suitable clothing for the poor, and dainty odds and ends which go to make a successful sale, cushions, knitted things, linen and work bags, all will be helpful. Some useful prizes are being offered, and particulars of the competitions will be found in the October number of *Nursing Notes*.

### A NURSING HOME AT HESSLE.

A "Nursing Home" has just been opened in Hessle, a village near Hull, as a development of a Cottage Nursing Association started for the surrounding districts some six years ago. A small house has now been taken, to which patients may be moved and properly nursed in cases where bad ventilation and drainage, combined with large families, prevent proper recovery in their own homes. Four beds are available, and the Home has been almost entirely furnished by gifts from kind friends. Two of the association nurses will live at the Home. A general and a house committee have been formed, and also a working men's committee, the villagers themselves being interested in the scheme which will be entirely supported by the inhabitants of Hessle. Mrs. F. R. Pease, whose husband is treasurer of the Hull Royal Infirmary, is its president.

### HULL DISTRICT NURSING ASSOCIATION.

The Hull Board of Guardians, on the suggestion of Dr. Jackson, have passed a resolution to subscribe "to the funds of the Hull District Nursing Association the sum of £5 per annum," subject to the approval of the Local Government Board. Dr. Jackson spoke very warmly of the "amazing excellence" of the work of the association, and, in spite of the very uncalled-for objections of one guardian, who said he "had seen plenty of nurses" and knew they were "a lazy, idle lot of beggars" (a statement needing no comment here, but which we are glad to see called forth strong reprobation from the meeting), when the question was put to the vote there were only four dissentients. The nurses of the association are all fully trained, and two of them are "Queen's Nurses." During 1895, 7,438 visits were made on 233 cases, and as many of the patients were in receipt of parish relief, the justice of a grant from the Guardians is obvious. The committee of the association are very anxious to engage another nurse for the heavy work of the winter, but funds are scarce, and more help is needed to make this increase in the staff possible. After Dr. Jackson's testimonial to the Hull nurses such help ought to be quickly given.

### BIDEFORD HOSPITAL AND NURSING SOCIETY.

The annual carnival and parade of the Bideford Cycling Club has just taken place, and the funds of the hospital and the nursing society benefited thereby to the extent of nearly £30. The Volunteer Band headed the procession, and the various entertainments went off in the most successful manner. In the evening the quay was illuminated with fairy lamps and coloured lights, and a promenade concert and dance were held in the market.

### CITY OF LONDON UNION INFIRMARY.

The *Daily Mail* of the 21st inst. states : At a general meeting of the Board of the City of London Union Infirmary, Bow Road, held on Tuesday, a resolution was adopted, by the casting vote of the chairman, that the Local Government Board be informed that the medical officer, Dr. Buncombe, and the matron, Miss S. A. Warburton, had ceased to retain the Guardians' confidence.

### ROYAL SEA-BATHING INFIRMARY, MARGATE.

The patients of the Margate Royal Sea-Bathing Infirmary enjoyed a musical treat lately, the children's choir from the Princess Alice Orphanage, New Oscott, Birmingham, on their way to Ramsgate with their superintendent, Mr. Thomas Durley, stopping for an hour or two to sing in the wards. Several children from the Orphanage have benefited by treatment received at the infirmary, and this was by way of a grateful return for the care bestowed upon them. After giving a number of glees and part songs the matron provided the members of the choir with refreshments. Another concert was held in the dining hall last month, got up by several of the infirmary's kind friends, and was a great success.

### WORKHOUSE NURSING AT TIVERTON.

A lady on the Tiverton Board of Guardians is endeavouring to effect improvements in the nursing arrangements of the workhouse, which appear to be very greatly needed. At present it is stated that the proportion of nurses to patients is one to thirty-seven. We hope Mrs. Wyndham will be well supported by her fellow workers on the board, and will succeed in securing the required increase in staff and the abolition of pauper help in the care of the sick inmates of the workhouse.

### SOUTH MOLTON NURSING ASSOCIATION.

Lady Susan Fortescue presided over the annual meeting of this association the other day. The report testified to a satisfactory year's work. The present nurse is popular amongst her patients, and it had been found possible to increase her salary. The financial statement presented showed a balance in hand of £10 9s. on the year's working.

### FORFARSHIRE CHARITIES.

The charity demonstration recently held at Brechin resulted, after paying all expenses, in placing a balance of £65 5s. 9d. at the disposal of the committee. It was decided to give £45 5s. 9d. to the infirmary, and the remaining £20 to the Victoria Nursing Association. The Dowager Countess of Dalhousie sent a donation to the Charity Committee.

### SALE OF WORK AT BRISTOL ROYAL INFIRMARY.

The Duchess of Beaufort opened a sale of work at the Bristol Royal Infirmary during the first week in October, having for its object the provision of new cots for the children's ward, and to raise a fund for sending small patients away to convalescent homes after leaving the infirmary. The stalls were arranged in the board-room, presided over by Miss Smith, the matron, and members of the nursing staff. Refreshments were served in the long corridor, which was prettily decorated with flowers and plants. There were a number of visitors during the afternoon, and the proceeds of the sale altogether amounted to £55.

### NURSING IN LABRADOR.

Sisters Carwardine and Williams, who have been so long connected with the work of the Mission to Deep Sea Fishermen out in Labrador, are still pursuing their labours at the Battle Harbour and Indian Harbour hospitals, and are likely to have heavy times during the coming winter, for the fisheries have this year been sad failures, and great will be the distress and probable consequent illness in the bitter weather to come. Drs. Grenfell and Willway have written pathetic accounts of "the bitter griping poverty" around them, and of the deaths that have occurred among the poor fisher folk from sheer starvation in such bad seasons. The medical help afforded by these brave workers brings untold comfort to many. Anyone wishing to know more of the M.D.S.F., and how they may themselves help forward its work, should write to the secretary, 181, Queen Victoria Street, E.C.

### MEMORIAL COTTAGE HOSPITAL AT JEDBURGH.

A successful bazaar was held at Jedburgh on October 6th and 7th, in aid of the Endowment and Furnishing Fund of the Sister Margaret Memorial Cottage Hospital. The site for this new hospital was presented by the Marquis of Lothian, and the cost of building has been defrayed entirely by Mr. A. Macmillan Scott, of Pinnaclehill, in remembrance of his sister, whose great wish had been the erection of such an institution for the benefit of the poor amongst whom she had spent her life. The bazaar was opened on the first day by the Marchioness of Lothian, and on the second by the Countess of Dalkeith. With the addition of a special donation of £100 the proceedings resulted in a profit of nearly £600.

### SHORT ITEMS.

The October meeting of the Association of Registered Medical Women was held on October 6th inst. at the New Hospital for Women, Euston Road. The President, Mrs. Garrett Anderson, took the chair, and twenty-two members were present.—The Superintendent of Nurses at the Plymouth Workhouse Infirmary informs us that two of her staff, Nurses Maxwell Moffat and Cave, have just successfully passed their examination for the L.O.S. diploma. Both nurses belong to the Workhouse Infirmary Nursing Association.—It has been arranged by the Guardians that a proportion of the nurses at the Wandsworth and Clapham Infirmary shall be provided with lodgings outside the building until such time as proper quarters are erected for the nursing staff.—The bazaar recently held in aid of the Women's and Children's Hospital at Cork resulted in a profit of £1,500. A debt of £500 still remains to be cleared off.—The private nurses attached to the Bristol District Nurses' Society (Berkeley Square) are permitted to undertake "visiting nursing," a convenient arrangement of which many patients are glad to avail themselves.—Plans for a new nurses' home for the Reading Workhouse Infirmary have been submitted to the Local Government Board, and by that body referred back to the Guardians for certain alterations.—Miss Jean Chapman, Miss Sydney Stanistreet, Miss Sophie Rhind, and Miss Constance Jacob have been admitted into the Order of Red Cross Sisters, Dublin, having passed satisfactorily through their hospital training and the required examinations.

### Hygiene: For Nurses.

By John Glaister, M.D., F.F.P.S.G., D.P.H.Camb., Professor of Forensic Medicine and Public Health, St. Mungo's College, Glasgow, &c.

### XXIX.—CLOTHING IN TEMPERATE CLIMATES—OF CHILDREN—AND OF THE SEXES CONSIDERED GENERALLY.

As was stated in the previous paper, woollen undergarments are an absolute necessity in a climate like ours, and for summer, as for winter, for the reasons already given. The objections urged against their use are usually: (1) personal discomfort, and (2) effeminacy. It is true that certain hypersensitive skins cannot comfortably bear the friction of ordinary wool, but for these the finer vicuna wool acts as an excellent and comfortable substitute. The second reason is urged only by those who are ignorant of physiological law, and it is one which ought to be combated whenever it is put forward. For those persons who are not robust, those who are predisposed to rheumatism or chills, those who are convalescent from illness, and for the infirm, they are indispensable; and for those who enjoy ordinary health they act as preventives of illness.

Clothing of Children.—Very erroneous opinions are entertained by many parents respecting the clothing of children. These have their origin in the desire to rear "hardy" offspring. While the desire is praiseworthy, the modes adopted to effect it are entirely wrong. It is pitiful to see children of well-to-do families imperfectly clothed as to their limbs, and, in addition also, as to the upper part of their bodies. Children are peculiarly sensitive to temperature changes, and bear the loss of body heat very badly, with the consequence, when clothed as indicated, that they are more liable to contract illnesses. The equilibrium between heat production and heat dissipation is easily disturbed in their case. The safest underwear for children is composed entirely of wool, and it ought to cover the trunk and limbs of the body almost entirely. This applies equally to children of both sexes, and to all seasons of the year. An objectionable habit commonly prevails of differentiating between the underwear of a female and that of a male child. This is unwarranted by nature, and is unphysiological. It is apt, too, to be perpetuated and accentuated as the girl grows into the woman. This must be protested against; for the difference in the bodily functions of the sexes demands greater protection for the trunk of the female body than of the male. Under-vests which are usually sleeveless, and which leave the upper part of the chest entirely unprotected, are but too common, but they are none the less harmful. Much has been made of late years of the substitution of "flannelette" for wool. Probably cheapness has had to do with its popularity. But when it is remembered that it is made solely of cotton, loosely wound and woven, it is not safe, where perspiration is a bodily habit, for reasons already given.

Let us now consider in what respects our modern modes of dressing are compatible or incompatible with health. Beginning with the head-gear, we find certain errors ready to hand. Fashion is largely to blame for these. The airy creations of the milliner may be "things of beauty," but in many cases they do not contribute to "joy for ever." Nature has compensated the gentle sex for the absence of adequate artificial head covering by providing an ample natural covering. This, probably, in a large measure, results from the airiness of the artificial covering permitting the hair to grow more luxuriantly. When, however, a woman comes to the shady side of forty years, penalties are often the result of a continuance of this fashion in the form of headaches, neuralgias, &c., which might be prevented by a judicious addition of less permeable material to the head-gear. Male head-gear also calls for criticism. Hard hats, undoubtedly, do not contribute to the comfort of the wearer for two reasons, viz., want of adequate ventilation, and compression of the arterial blood vessels of the scalp at the fitting parts. There is little doubt that baldness is a common result from constant use of such hats, and from these causes much of the increasing baldness arises. That such hats are not worn from choice is shown by the alacrity with which, upon opportunity, they are cast aside in favour of head-gear of a softer and more pliant type.

Body-clothing.—Compatible with health, and the relative functions of the sexes, it cannot be gainsaid that the body-clothing of the male is more suitable than that of the female. In the former the trunk and limbs of the body are more uniformly covered, and there is less constriction of the trunk than in the latter. In the female the arms and upper part of the chest are imperfectly protected, and the lower part of the trunk is over-covered. The mode of suspending the lower garments, too, is decidedly objectionable, as an unequal strain is put on the muscles of the back and abdomen. Considerable advantage would result if part of this weight could be suspended from the shoulder, after the manner of male attire. The "rational dress" movement is a distinct advance upon the older mode of clothing. Much has been written against the corset, and probably its general use has been too much condemned, but it will be a necessity so long as the undergarments are suspended from the trunk. At the same time, not a word too much has been said against "tight lacing," which is provocative of much internal mischief, and tends to cripple healthy motherhood. By the substitution of lighter, but equally warm skirts, and by the use of the divided skirt, the corset need no longer remain of the unyielding character it possesses.

Foot-gear.—Here, also, some measure of reform is needed. Hitherto the anatomy of the foot would appear to have been totally neglected. Indeed, we take much better care of the feet of our horses than of our own. The pointed boot—that which tapers to a rounded point—like the very square-toed boot—is based upon a total misconception of the shape of the foot. A happy medium between the two is what is needed, a boot which, in other respects also, fits the foot as a glove does the hand, and which enables the foot to perform its various movements comfortably. High-heeled boots are also to be condemned. Nature intended that man should walk on the sole of the foot, not upon the heel or the toes, and that the foot should equally bear the body weight. The curves of the spinal column were intended for this purpose, but the high-heeled boot, in throwing the weight of the body on the front of the foot, distorts the proportion of these curves. To satisfy hygienic needs, the boot ought to be fitted when the foot is placed on the ground supporting the body weight, and shaped in front as the foot is shaped. It should possess a broad heel situated under the heel of the foot, and of sufficient height to enable the foot to bear equally the weight of the body, and it should be capable of ventilation. This last point is difficult to attain, for where air can get out damp can come in, and modern polishes do but help to clog the pores of the leather; hence ventilation only occurs at the lacing, or at the gussets in elastic-sided boots. Much has been urged against the use of gutta-percha for boot soles. In our view much of the criticism is only theoretical. Experimentally we have used boots the soles of which are made of alternate layers of canvas and gutta-percha, and have found them equally as comfortable as leather-soled boots. They exclude damp better than leather, but they are not quite so yielding as leather. Were it not, indeed, for this objection, the "clogged" boot—the boot with wooden sole—is about the healthiest form. Special attention ought to be given to the foot-gear of children during the period of growth, as boots then very quickly become too small, from which deformed feet, or, at least, bunions, callosities, or corns may result.

Dress for Nurses on Duty.—The dress of the nurse ought to combine cheerfulness of colour-tone, personal comfort to the wearer, ease of washing, and be made of a material which has the minimum liability to carry septic or infective matter. Cotton fabrics of fast colour, therefore, combine these conditions better than any other material, for they can be boiled and ironed, or pressed frequently without harm to the fabric, which cannot be said of woollen gowns. Made plain, and presenting a glazed surface from the pressing, they afford a less suitable resting-ground for microbes than do woollen ones. In nursing infective or septic cases more than ordinary precautions must be taken. A linen, thin *rubber* overall is very useful, as it can easily be put aside or put on on leaving or entering the sick-room. A nurse should never be allowed to pass from an infective atmosphere to the outside before her nursing uniform has been changed, a bath has been taken, and special precautions made in respect of her hair. Hitherto, too little attention has been given to the hair as a medium of carrying infection, and while we scrub and polish, and asepticise our hands and arms, we are too apt to forget the toilette of the hair. The hair of the head and face of the physician or surgeon, and the hair of the head of the nurse have been rightly blamed as the occasional vehicles of contagion of puerperal fever; therefore the hair ought to receive special attention. By wearing a loosely-fitting, ventilated cap of thin rubber, much after trouble might be prevented. If, in short, the "preventability" of preventable diseases is to become an accomplished fact, it is only in some such manner as the foregoing that this end will be secured.

### Mediæval Practice in the Care of the Sick.

II.

We find that although many religious orders existed in mediæval England, there were not anything like the number of societies devoted exclusively to the care of the sick as are to be seen in operation among English Catholics to-day.

On the other hand, in times when intercommunication between town and town, or between city and village, was difficult, housewives were of necessity often thrown upon their own resources in cases of emergency. The consequence was that gentlewomen in castles, nuns in convents, and "wise women" among the poor, accumulated a considerable amount of knowledge which stood them in good stead in the absence of physicians. Indeed, for the practical curing of common diseases by simple remedies, these women were generally of greater service than learned doctors, who discoursed gravely upon the conjunction of Saturn with Mercury, or the combined planetary influence of both upon the "humours" of the patient.

A handbook of nursing written in 1886 would hardly meet the requirements of an enthusiastic probationer in 1896, but no such analogy holds in old England. Galen, Hippocrates, and Aristotle were the medical authorities directly or indirectly studied by professors of the healing art, from the days of Alcuin to those of Linacre, and physicians who disdained to notice small-pox because the disease was not described by a Greek master could hardly expect to be seconded by nurses who "advanced."

Family traditions favour conservatism; the recipe books, containing directions for brewing simples, or concocting remedies for burns, scalds, fevers, and wounds, were doubtless handed down from mother to daughter, and from one generation of religious in a convent to another, so that, although there might be growth of experience and gradations of knowledge—true and false—for all practical purposes we may deal *en masse* with the art of nursing from Saxon days to the revival of learning in the sixteenth century.

The Greeks were regarded as the masters of medical lore, but perhaps in nothing does the contrast between Hellenic and Romantic ideals come out so sharply as in the manner of regarding humanity's fleshly envelope.

To the beauty-loving, joyous Greek a man's body was as divine as his soul. Galen calls the body a hymn praising God; and it may be doubted whether the Hellenes were capable of contemplating a state of happiness in which the two were separate. Homer's blood-drinking shades are wretched beings enough. It was for the sight of Helen's outward form the Trojan elders said it was good to die; ugliness of body was indeed so connected with evil in the Hellenic mind, that the sculptors represented Pericles wearing a helmet to avoid exhibiting the faulty shape of that statesman's head.

A very different note is struck in our mediæval literature. Everywhere the flesh is regarded as man's worst enemy; little attention is paid to the body in health; sickness is a most useful mortification; and monk-physicians, even in teaching remedies for the evils to which humanity is heir, do so with apparent searchings of heart, interspersing their remarks with admonitions more befitting their clerical than their medical capacity.

A poem on Death, probably written during the reign of Henry III., and which has been printed by the Early English Text Society from the Jesus College MS., describes, for instance, how "as soon as the soul is gone out of the body" it is sown in "a clout," and how this form of humanity, that was once "so nody and strong, and so very proud, wont to wear many a fair shroud" during life, will nevertheless become corrupt. Its limbs will rot, and worms will feed upon what will soon be an object of terror to whiledom friends; the moral follows, describing the vanity of "all thy rich wearing," and thy many servants to wait upon that which is doomed so quickly to perish.

A painfully realistic account of the "Signs of Death" also points to the same moral, when all is over with a man's life . . . "his eyes shallen dymnen. . . . His skyn shal starken . . . and his tonge shal stameren . . . and thou that art proud ne shalt thou have but a Clout, to cover thy dead body, which will be hurried out of sight by the survivors as a loathsome thing."

These may be—indeed, are—noble sentiments, but administered in large quantities they materially affected the mediæval view of sickness.

### Presentation.

A pleasant musical entertainment was given at the Hull Sanatorium on Tuesday, October 5th. During the evening Mr. Alderman Fraser, Chairman of the Sanitary Committee, on behalf of Miss Bland, the Lady Superintendent, and the nursing staff, presented Nurse Burgoyne, who is just leaving the sanatorium, with a writing case as a parting gift. Nurse Burgoyne expressed her thanks through Dr. J. Wright Mason, the Medical Superintendent. She has been four and a half years at the sanatorium as probationer and assistant nurse, and has now joined the staff of the new Brook Hospital, Shooter's Hill.

### Nurses in 1896—Their Quarters, Hours, and Food.

[These articles exhibit the actual condition of affairs in the spring of the present year.]

### ROYAL FREE HOSPITAL.

### I.—Terms of Training.

The Royal Free Hospital has followed the example of St. Bartholomew's in requiring ordinary probationers, upon entering its service, to sign an agreement for four years instead of the more customary three. Candidates are eligible between the age of 23 and 35, and are admitted on trial for three months. At the end of this time, if considered suitable, the agreement is signed to serve the hospital for four years, three as probationer, and for the fourth year on such duty as may be prescribed by the matron, the service dating from the day of entering the hospital. Medical and surgical lectures are given, which probationers are required to attend, and the certificate of the hospital is granted at the end of the four years' period of training to those who have passed the prescribed examinations and are approved by the Weekly Board.

A certain number of paying probationers are received, who must be between the age of 23 and 45 years. They sign an agreement to serve the hospital for six months, during which time their work is the same as that of the ordinary probationers. They are allowed to attend the lectures and benefit by the technical instruction given to the ordinary probationers. The fee is thirteen guineas for each period of three months, payable in advance, and special probationers are required to pay their own laundry expenses and to provide themselves with the uniform of the hospital. If at the end of the six months special probationers desire to join the regular nursing staff, and are recommended to the board by the matron, they can do so upon signing an agreement to serve as ordinary probationers for the rest of the period of four years, that period dating from their first entrance into the hospital.

### II.—Hours of Work and Times off Duty.

The nurses and probationers are called at six a.m., going on duty in the wards at seven a.m., and leaving them at half-past eight p.m. During these hours two hours off duty are allowed daily, the ordinary times on week-days being from ten a.m. to twelve noon, two to four p.m., or four to six p.m. On Sundays, ten a.m. to one p.m., two to four p.m., or two to six p.m. The rules state that "as far as is practicable," nurses are off duty one evening in the week from six to eight. Night nurses are on duty from nine p.m. to nine a.m. On week-days they have their off-duty time in the morning, from ten to twelve; on Sundays, in the evening, from six to nine. Sisters come on duty at eight, and leave the wards at night at half-past nine, being expected to enter in the "time book" kept at the entrance to the nurses' quarters the time they leave the wards each night. Their off-duty time is six to eight p.m. Twenty minutes is allowed the nurses for dressing after the heavy morning's work, and for dinner and tea twenty minutes to half an hour, so that hours actually in the wards are approximately a little under ten.

In addition to the daily leave, one whole day, from seven a.m. to ten p.m., is accorded to all the staff each month, and once a month also leave from six to ten p.m. A late pass is given once in three months from six to half-past eleven. Nurses on night duty have two nights off duty during each three months. Day nurses have to be in their dormitories by ten p.m., and in bed by half-past ten, so that seven and a half hours in bed are secured to them. Night nurses are required to be in their dormitories on week-days by twelve noon; on Sundays by ten a.m. Night and day duty is taken in alternate periods of three months. There are no permanent night nurses at the Royal Free Hospital.

Very strict punctuality is exacted from the nurses, and they are required to enter their names in the "time book" when returning from off duty. All nurses are required to go out for exercise when the weather permits during their time off duty unless excused by the matron. . . . Night nurses are so excused on Mondays and on the day after their long day. The printed rules are stringent in many particulars. Especially may be noticed that nurses who are late at breakfast more than once will be liable to forfeit their extra leave at the matron's discretion. Also no nurses are allowed to receive male visitors except by express permission of the matron, and when on duty in the wards no other needlework save such as the sister may direct, or, with the sister's permission, making up of uniform caps and aprons is permitted, but no reading or writing.

### III.—Meals.

The day nurses' breakfast is at twenty minutes to seven a.m., the second breakfast for the sisters following at half-past seven. For this meal fish, bacon, or eggs is provided, with tea and coffee. Luncheon of bread and butter and coffee is taken in the wards. Dinners are at twelve to half-past twelve, and one to twenty-five minutes past one. Tea is laid in the dining-room from four to a quarter to five p.m. Supper is at eight for sisters and junior probationers, who return to the wards for half an hour afterwards, and at half-past eight for senior nurses and night nurses. Night nurses have their dinner at nine to half-past nine a.m. The night meal is taken from supper to the wards, usually packed in baskets, and consists of fish, cold meat, eggs, &c. Bread and butter is taken fresh from the dining-room each night, tea and sugar being the only stores given into the nurses' hands twice a week.

For dinners hot meat and vegetables, puddings or tarts, are given, with a certain amount of beer and an unlimited supply of milk. At tea, in season, watercress and radishes are provided, and jam, occasionally biscuits. For suppers cold meat, potato pies, fish pies, hash, and puddings. Fish is always given on Fridays, with cold meat for those who prefer it.

### IV.—Salaries and Uniform.

No salary is given to probationers during their first year. They are paid £15 for the second year, £20 for the third, and £25 for the fourth. The salary of nurses who have completed their four years' training is £27 per annum, increasing annually £2 to £30, or until reaching the position of sister. Sisters' salaries commence at £30, increasing £2 annually, till reaching £40. The Royal Free Hospital is affiliated to the Royal National Pension Fund for Nurses, the committee paying one half of the annual premiums of all those certificated nurses in its service who may desire to join.

Probationers on trial are required to provide themselves with uniform for these three months, the amount prescribed being three dresses, twelve aprons, and three caps, similar to the uniform of the hospital. When accepted on the staff of the hospital, probationers are allowed, during the first year, materials for one dress, one cap, and eight yards of linen for aprons; during the rest of their service materials for three dresses, three caps, and eight yards of apron linen per annum, while an allowance is made to cover the cost of making up the dresses.

### V.—Nurses' Quarters.

The accommodation allotted to the nursing staff at the Royal Free Hospital badly needs improvement and additions. A portion of the easternmost block of the hospital is devoted to this purpose, and provides cubicles only, alike for sisters, nurses, and probationers. The dormitories are light and airy, and look as comfortable as possible under the circumstances, but the cubicles are very small, and possess to the full the usual disadvantages of such sleeping places. The presses for clothes have to find room down the centre of the long dormitories. A few of the senior sisters have small bedrooms to themselves, cosily furnished; but for the rest, as we have said, their accommodation is the same as the probationers. There are no sisters' rooms off the wards at this hospital. There are two pleasant sitting-rooms, one for sisters and one for probationers, provided with comfortable sofas and easy chairs, and the dining-room in the basement is not an uncheerful apartment, in spite of its underground situation. There is but one for all the staff. Baths, too, are limited, two only existing for the use of all the staff. It is certain that the next work taken in hand at the Royal Free Hospital should be the provision of better quarters for the nurses.

### Midwifery Papers.

### VIII.—SOME COMPLICATIONS OF LABOUR.

Twins.—The chief guide before labour in the diagnosis of twins is the large size of the abdomen. Sometimes in labour one head is found presenting, while another can be felt at the fundus, but very often the first child is born before the presence of the second is discovered. The uterus remains large after the expulsion of the first child, and the abdominal walls are distended. Twins can present—one in the breech and one in vertex position; or two breech, or two vertex. One breech and one vertex is the most common. Owing to the extreme distension of the uterus the labour is generally prolonged, especially if the first child presents by the breech. After the birth of the first child the pains subside to recommence about ten or twenty minutes later. The second child is more rapidly expelled, owing to the previous dilatation of the soft parts. When the first child is born the cord should be tied and cut in the usual way. Generally in twin pregnancies there is one placenta with two cords and two separate bags of membranes. If the presentation of the second child is normal, the management of the labour proceeds in the usual way. Assistance must be at once summoned if the second child should present in the transverse position. Great care must be taken after the birth of twins to ensure firm contraction of the uterus; uterine inertia may result after the great distension, and there is considerable danger of hæmorrhage.

Still-birth.—When the second stage of labour has been very prolonged, as in breech or face presentations, and the child's head has been subjected to much pressure in its passage through the pelvis, it may be born apparently dead. The cord must be at once tied and separated, the nose and mouth cleared of any mucus, and respiration excited by stimulating reflex action. Sharply flicking the back and chest with a damp towel, or plunging the child alternately into hot and cold water, are remedies which should be tried at once. If you suspect that the bronchial tubes are blocked with mucus, try to empty them by holding the child by its feet head downwards, meanwhile rubbing briskly up and down its spine. Sylvester's method of inducing artificial respiration is also recommended. No time should be lost after the birth in the attempt to make the child breathe. If your efforts do not succeed at first, still persevere, for cases have been known in which respiration has been induced after the lapse of a considerable time.

Macerated Fœtus.—If, when the membranes are ruptured, the liquor amnii is very fetid and dark-coloured, you may suspect the presence of a dead fœtus. The absence of the fœtal heart-sounds and movements will confirm this. If the fœtus has been dead some time it will most likely be expelled in a decomposed condition. The chief causes of the death of a fœtus in utero are: (1) Fright or accident to the mother, (2) diseased placenta, (3) syphilis, (4) asphyxia from pressure on or prolapse of the cord or ante-partum hæmorrhage. Very often when the fœtus dies it acts like a foreign body on the uterus and sets up contraction, labour is induced, and it is discharged. But a dead fœtus may be retained some time, and labour may come on at the expected time. The chief complication in this labour is the increased risk from hæmorrhage and blood-poisoning, owing to the unhealthy condition of the uterus and placenta. The placenta should be most carefully examined after it is expressed for in its diseased condition it is more easily broken up and portions may be left adhering to the uterine wall. Extra precaution should be taken to cleanse the parts with an antiseptic douche and to guard against hæmorrhage.

Rupture of the Uterus.—This is one of the most dreadful complications in midwifery. It may be caused by (1) excessive contractions of the uterus induced by the administration of ergot in the early stages of labour; (2) by strong pains with some obstacle preventing delivery; (3) by diseased, uterine walls. The symptoms of rupture are a sudden and excruciating pain accompanied sometimes by an audible snap. Sometimes the fœtus escapes from the uterus into the abdominal cavity, and the presenting part recedes and can no longer be felt. There may be considerable hæmorrhage from the vagina, and the patient exhibits the marked signs of internal bleeding and collapse in the blue drawn face covered with cold clammy sweat, quick feeble pulse, hurried respirations, and vomiting. This complication generally terminates in the death of both mother and child. If there are strong pains with no advance of the presenting part, and the vagina is normal in appearance, the midwife must conclude that there is some obstacle in the pelvis or on the part of the child to delivery, and she must at once send for a doctor. Remember that strong uterine contractions are a danger if any obstacle is present, and may result in the dreadful catastrophe—rupture.

Pelvic deformities complicate labour very seriously. They are very difficult to diagnose, but often if the pelvis is contracted the presentation is not natural, and the midwife's duty is to recognise this and send at once for assistance. If a doctor has to be summoned the midwife should remain and give him all the help she can. He may have to deliver with instruments; if so, have plenty of hot water and antiseptics in readiness. Remember, when a presentation is transverse, the doctor has generally to perform "turning," that is, to change the position of the child into one in which it can be born. This is done best just after the rupture of the membranes, and when the parts are as fully dilated as they can be; so keep the membranes intact if you can till he arrives. Unfortunately, in pelvic contractions, rupture of the membranes spontaneously occurs very often early in the labour, and the liquor amnii dribbles away, the dilatation of the os is delayed, and the delivery becomes even more difficult. In any case of delayed or complicated labour conserve the strength of the patient as much as possible. Do not allow her to waste her strength by over-exertion in talking or moving about, and persuade her to take light nourishment at intervals. If you want to keep the membranes intact do not examine too frequently, and keep your patient in a recumbent position.

Protracted Labour.—"Natural" labour is said to begin at "term," and end within 24 hours without interference; but in most cases the length of labour varies. In first cases there is slower dilatation of the parts now stretched for the first time, but when real (as distinguished from false) pains set in the labour is generally over in 24 hours. Labour is generally shorter in women who have previously borne children, and ends in six or eight hours. But the uterus is very much under the influence of mental conditions, and in highly nervous women labour may be delayed by nervous excitement. In cases of protracted labour the nurse should satisfy herself that no cause of uterine inertia which she can remove (such as loaded rectum or bladder, extreme uterine distention from excess of liquor amnii, &c.) is present. If the delay continues, after due precautions have been taken and remedies tried, and especially if the membranes have been ruptured, the nurse should summon assistance, for in uterine inertia the risks are many to both mother and child, and it may be necessary for the doctor to deliver with instruments. Do not wait until your patient becomes worn out by the ineffectual pains, and her pulse and temperature begin to rise, before you summon assistance.

### Royal British Nurses' Association.

SIR J. CRICHTON BROWNE ON HIS ASSAILANTS.

Her Royal Highness Princess Christian presided at a meeting of the General Council of the Royal British Nurses' Association held on Friday, October 16th, at 17, Old Cavendish Street. Thirty medical men and thirty-nine matrons and nurses were present, amongst them Sir James Crichton-Browne, Sir Dyce Duckworth, Mr. Pickering Pick, Dr. Outterson Wood, Miss Thorold, Mrs. Dacre Craven, and Miss de Pledge. The reports of the hon. treasurer and the Executive Committee were adopted. In commenting upon the financial condition of the Association, Mr. Langton deplored the enforced expenditure of the funds upon litigation, which had of late cost the Association no less than £257, and said he was glad to announce that recent subscriptions and receipts had reduced the present deficit to £116.

Dr. Outterson Wood then read the report of the committee appointed to consider the desirability of admitting mental nurses, as such, to registration and membership of the association. The committee were of opinion that such a step would be desirable under conditions which they indicated. The adoption of the report was seconded by Sir James Crichton Browne, and duly carried. A conference with the medical superintendents and matrons of asylums will be the next step taken in this matter.

With the permission of the President, Sir James Crichton-Browne, in relation to his resignation of the office of vice-president, then gave the following explanation.

Sir J. Crichton Browne on the Defensive.

As I daresay most of you are aware, during the last fortnight an action has been brought against me in the City of London Court—that is to say, the County Court for the City of London—by Miss Breay, a member of this Association, on the ground that I had maliciously and wrongly, as she alleged, in my capacity as chairman, refused to put to the annual meeting a resolution proposed by her. Miss Breay claimed £50 damages and got one farthing. I think some explanation is due to the Council and to myself as to the manner in which that action arose and was conducted.

Among the other flattering insinuations made at the trial it was suggested that the keeping back of the resolution was a pre-arranged conspiracy between me and the other officers, and that we did it to shelter ourselves from Miss Breay's censures. I say such an insinuation is false and calumnious. Mr. Langton swore that the officers were anxious to have the resolution taken, and I did the same; and, whatever my failings may be, I am not quite an absolute idiot. The resolution was, as I have said, a sweeping unmeasured condemnation of the Executive Committee, of which I am myself a member. It had been advertised. It had been circulated. It was in the hands of everyone. There had been no attempt at suppression. It was surely advisable that it should not go unanswered when its egregious misrepresentations could have been readily exposed; least of all was it desirable that it should be left unanswered at a meeting at which there was a large majority supporting the Executive Committee. Miss Breay herself swore that she did not expect her resolution to be carried. Dr. Bedford Fenwick had been lamentably defeated in his attempts at obstruction at that meeting. There could be no doubt of the issue had the resolution been taken. I well knew the temper of the meeting. It was no fear of the result that led me to rule the resolution out of order. I will tell you my real motive. It was to avoid litigation. Again and again have I heard the management threatened with legal proceedings at the Executive Committee. Last year, as you know, the Association was involved in a very costly lawsuit by Miss Barlow; this spring, the late Sir Russel Reynolds was, greatly to his distress, when on his death-bed threatened with legal proceedings by these same solicitors who brought this action against me. I felt tolerably sure there were persons at the annual meeting, lying in wait for some fresh pretext for another action; and I judged that the only way to deprive them of their opportunity was to adhere to the strict letter of the law,

The question was put to me, and I was bound to decide then one way or the other; there was no middle course. Now, suppose I had decided the other way, and, ignoring the bye-law, had allowed the resolution to be put. If it had been carried it would have been open to any member of the Executive Committee who was censured by it to object to the result, because it had been brought forward in defiance of the bye-law. If it had been lost it would have been open to Miss Breay to say we had forced on the resolution in defiance of the bye-law because we found we had a majority at the meeting, a number of her supporters having gone to view the Royal wedding, and that, had we postponed it, as we ought to have done, until another meeting, there would have been a very different result.

To avoid litigation, to adhere strictly to the bye-laws, I refused to allow to be put a resolution which had not been sent by registered letter, and which was in itself highly irregular, for, as you will notice, it sought to condemn the present Executive Committee for transactions for which previous committees were responsible, and it raised again the case of Miss Barlow, which was finally disposed of at a special general meeting summoned by Her Royal Highness the President last January.

Well, I refused to allow the resolution to be put, and what took place then? Why, Dr. Bedford Fenwick rose and said he must bow to the chairman's decision. This gentleman, who is found a little later countenancing, supporting, if not instigating legal proceedings, against the chairman, bowed to the chairman's decision.

Only one other fact in connection with the meeting will I refer to, and I do so merely to illustrate the purposely misleading character of the reports of the proceedings of the Association purveyed for nurses in that veracious, I had almost said ferocious, periodical, the *Nursing Record*, that very appropriately has a pink outside, as if blushing for its own transgressions. In its report of the annual meeting there occurs the following: "A vote of thanks was moved to the chairman, Sir James Crichton-Browne, and, having been duly seconded, was protested and voted against, but declared carried." What is the obvious inference from that statement? That there was a narrow division in the vote of thanks to the chair; that the result was even doubtful; but that it was "declared carried." But what are the real facts? It was Mrs. Bedford Fenwick who graciously opposed the customary vote of thanks to the chair, and when that vote was put to the meeting by Mr. Langton, the treasurer, Mrs. Bedford Fenwick was supported by exactly five persons. In a meeting of over one hundred and seventy, five supported Mrs. Bedford Fenwick. I could give you many instances of the same sort of thing in the *Nursing Record*, and I do think it is desirable that it should be generally understood in the nursing world that this *Nursing Record*, the Nursing Discord it should be called, is in no sense the organ of our Association, that we repudiate it and all its ways, and hope soon to have a trustworthy weekly journal of our own.

As Miss Breay's statement at the annual meeting that her letter really was registered had attracted attention, a few days later Miss Wilmot was sent to the Vere Street post office with the letter, to show it to the clerk in charge. She did so, and he unhesitatingly pronounced that it was not registered. Surely if a post office expert could not recognise it as a registered letter, a chairman of a public meeting may be excused for not having done so.

As soon as I learned that there were grounds for believing that Miss Breay's letter was registered, I wrote to the honorary secretary, suggesting that she should be offered a special general meeting, at which to bring forward her resolution, and this was, I believe, done with the sanction of your Royal Highness; but Miss Breay was then in the hands of her solicitors, and this did not suit their purpose, or the purpose of those who were instructing them. A scandal was wanted, and so the offer of a special meeting was declined. These vexatious legal proceedings were pressed on.

### The Trial of the Action.

I now come to the trial of the action against me, and upon that subject I shall not detain you very long. I was assured by the solicitor of the Association that the evidence required of me at the trial would be purely of a formal character, and on my hinting that possibly an attempt might be made to prejudice the case by dragging into it past controversies in the history of the Association, he said that would not be allowed. At the beginning of the trial the Judge remarked: "We cannot in this Court review the proceedings of this Association." But notwithstanding that he permitted the counsel for the plaintiff to survey its proceedings for the last two years, and to introduce what were necessarily partial and misleading versions of them. We went into court simply prepared to deal with the cause of action, and with what took place at the annual meeting, and we were, therefore, at a disadvantage. Had we had the least idea that all former controversies and differences were to be raked up, we should have gone with an overwhelming weight of evidence to rebut the statements made.

As regards the evidence of Miss Breay, the plaintiff, I will only observe that it was fair and moderate, and given without apparent animus. I have no doubt Miss Breay thought she had registered her letter, and that she felt aggrieved because her resolution was not taken, so that she could deliver the speech she had prepared; but I question very much whether, if left to herself, she would have resorted to legal proceedings before availing herself of those other means of obtaining redress which the constitution of the Association offers.

Of course much hinged on the question of the registered letter. The Judge laid it down as the law—and I would not for one moment dispute his ruling—that the essential parts of registration are the inscription at the Post Office and payment of the fee, and that the marks on the letter are only evidence of registration. Well, even on that view, I am still incredulous whether Miss Breay's letter was, legally speaking, registered. I heard Miss Breay state in the witness box that she paid three pence for the letter; but that is the charge for an express delivery letter; and had she registered it as well, she ought to have paid five pence. I heard Miss Giuseppi state in the witness-box, that the receipt she signed was for an express delivery letter, and that the word "registered" did not occur on it; and by a very curious coincidence Miss Giuseppi's receipt, when called for at the Post Office, could not be produced; it had been unfortunately mislaid. Not until I see that receipt with the word "Registered" on it and am satisfied that Miss Breay paid the full fee shall I believe that her letter was legally registered; and even then, even if satisfied on these points, I should unflinchingly adhere to my decision, for the point at issue was not, was the letter registered, but was it registered to my knowledge, when I gave that decision. It bore no evidence of registration, none of the ordinary and well-recognised marks. Am I to know a registered letter by intuition or instinct, or by subtle aroma proceeding from it? I have seen thousands of registered letters, but never one like this. I could not believe that the marks on an ordinary registered letter—the cross blue lines, letter "R," stamps, &c.—were superfluous, put in for the amusement of the Post Office officials. I regarded them, and do regard them, as essential to identification. They were not there, and so, to the best of my knowledge and belief, the letter was not registered, and I rejected it; and I should do so again under the same circumstances to-day.

### All the Controversies of the Past.

As I have told you, all the controversies of the past were raked up at the trial, and I was made responsible for everything that has happened. I had frittered away the funds of the Association and brought it into difficulties. As a matter of fact, I have had nothing to do with finance; and, if I am correctly informed, it was Dr. Bedford Fenwick who, when treasurer, spent the bulk of the funds—no doubt very properly—and resigned his office when they were all but exhausted and he saw difficulties impending. And in connection with finance there was one statement made by Dr. Bedford Fenwick on oath which I should be glad to hear him justify. He said the Association is now £800 in debt, whereas I heard him informed at the Executive Committee a few days before the trial by the treasurer that the total liabilities of the Association are about £400, and I saw him make a note of the fact.

But not only had I frittered away the funds of the Association, but I had excluded the matrons from the council. As a matter of fact, I was always opposed to the exclusion of the matrons from the council. Not only did I exclude the matrons from the council, but I was responsible for the Barlow case. As a matter of fact, the Barlow case was well advanced before I heard anything about it, and I did my best, as Dr. Bedford Fenwick knows, to put a stop to it.

### The Charge of Partiality.

Of all sorts of high crimes and misdemeanours had I been guilty. I shall not weary you by recounting them; but there were two charges made to which I must particularly allude, and these were that I was partial, and that I had entered into a conspiracy with five or six leading medical men to seize upon the whole power of the Association and deprive the nurses of their just rights. As regards the charge of partiality, I will merely say that when a chairman is called upon to decide any knotty point in a meeting in which feeling runs high, the party against which he decides generally pronounces him partial. Now it has been my lot, unhappily, to have to decide knotty points again and again in very stormy meetings, and I daresay my decisions have generally been against the noisy agitators whose methods I have regarded as irregular and unjust, and thus, I have no doubt, I have incurred their displeasure. But that was their fault and not mine; and had they always been in the right and conducted themselves in order, my decision would always have been in their favour. I can conscientiously aver that I have done my best under the most trying and perplexing circumstances to act with fairness and firmness, so that business might be despatched and the best interests of the Association protected. I was proposed for the vice-chairmanship by Dr. Bedford Fenwick, who has repeatedly complimented me on my impartiality and firmness; I believe he has called me a model chairman. Only last year, after the annual meeting of 1895, when I was trying to avert difficulties arising in connection with the Barlow case, he said to me "We are quite satisfied with your conduct in the chair. We have no objection to you. It is to others we object." Dr. Besley Thorne was, I believe, at that time the arch-enemy of mankind, or rather womankind, a position to which, since his retirement, I have been promoted. It is impossible to please everybody. When leaving the annual meeting this year I was blamed for being far too courteous and conciliatory to Dr. Bedford Fenwick and his party in their obstruction tactics, and for not putting them down in a much more peremptory manner than I did.

### The Charge of Conspiracy.

As for the charge of conspiracy with other medical men to seize on power and deprive the nurses of their rights, it is really too preposterous, and I must leave others to answer it. It is slightly inconsistent with Dr. Bedford Fenwick's other statements, for he has described the Association as a bankrupt concern. Is it not curious that medical men should be anxious to obtain control of an Association in such a position? There is generally a motive for a conspiracy. What is the motive in this case? You know very well that medical men have contributed the larger proportion of your funds, taken a prominent part here simply and solely from a desire to help you, with no selfish ends of their own to serve. You know that they have been forced into prominence simply from the reluctance of nurses to put themselves forward, especially in such heated and truculent scenes as Dr. Bedford Fenwick and his party have created. You know that they, the medical men, have had the constant co-operation and assistance of some real leaders in the nursing world, Miss Thorold, Mrs. Dacre Craven, Miss de Pledge, Miss Wedgewood, and others. For my own part, as many of you are aware, I have never ceased to urge nurses to take a more and more active part in the affairs of the society. There is not one of these medical men who would not willingly hand you over the whole of their affairs to-morrow, and it is a little too much of a good thing that men in their position should be accused of conspiracy to seize power, by Dr. Bedford Fenwick. It is a little ridiculous, too, that I should be accused at the trial of grasping at power when at this very annual meeting I had announced my intention of resigning office, feeling that I had borne my full share of work in connection with the Association.

### "The Small Discredited Faction."

But how about the other side, the small discredited faction? Are they, like Cæsar's wife, above suspicion? Have they passed a self-denying ordinance? Have they no ends to serve? Have they no lust for power?

This action, ladies and gentleman, to which I have been referring, is, as far as I myself am concerned, a very paltry affair. I can afford to view with equanimity the verdict of a county court jury of five and the contemptible damages of one farthing; but as it concerns this Council and this Association, it is a matter of great importance, for it is part of a general policy which has been doggedly pursued for some time past, a policy which aims at bringing this Corporation to the verge of ruin. You have seen that policy in operation, and you know its working. Its object is, by attacking officer after officer, to make their positions untenable, and to drive them from your service; by misrepresenting and insulting those leading members of my own profession who have interested themselves in you, and have tried to help you, to alienate them and force them to resign; by incessant litigation to cripple your resources; by holding you up to obloquy, as a bankrupt undertaking and as a hotbed of dissension, to frighten new members from joining your ranks; by incessant wrangling and obstruction to make the conduct of your business impossible; and, finally, by isolating and disgusting her, to induce your Royal president to abdicate the presidential chair. Disguise them as they may, these are the real aims and objects of the small and discredited section with which we have to deal. Defeated again and again in every open contest, they strive by indirect means to compass their end—which is to bring this Association to the last gasp—and then, when it is at its last gasp, this small and discredited section will seize on the charter and the Association, and exploit them for their own exaltation.

I cannot, perhaps, pretend to say exactly what might then happen, but very possibly you might have Miss Kenealey as president, Mrs. Bedford Fenwick as secretary, that great gifted financier, Dr. Bedford Fenwick, as treasurer, and that eminent physician, Dr. George Brown, as honorary medical secretary.

### Do You Like the Picture?

Do you think your interests (I am speaking to the nurses) will be as well safe-guarded then as they are now? Do you believe the public will regard your calling with equal favour?

If you like the picture by all means throw yourselves into the arms of the litigious agitators. Your medical representatives will, I am sure, on the slightest hint, willingly wash their hands of your affairs, though not without grief to see what should have been a splendid success reduced to miserable failure. But if you do not like the picture, if true to your instincts, to your nature as nurses, you wish to pursue the path of peace, order, and propriety, of forbearance and gentleness and courtesy; if you wish to preserve this Association intact, to see it grow and prosper and become a real shelter for nurses, then rally round your Royal president, support the officers whom you have elected, who have given of their money, their time, and their toil to help you; and say to the agitators in emphatic terms, from this hour your turmoils, your persecutions, your carpings must cease. In the words of the National Anthem, "Frustrate their knavish tricks, Confound them all!"

Dr. Bowles then proposed a resolution that Sir James Crichton-Browne's resignation be not accepted, and that he be re-elected vice-chairman for the ensuing year, which was seconded by Miss Thorold.

An attempt on the part of Dr. Bedford Fenwick to reply to Sir James Crichton-Browne produced such a demonstration of protest that the Princess put the question to the meeting if he should be heard, receiving an emphatic and unanimous answer in the negative, accentuated with strong expressions of indignation when it was announced that at that moment steps were being taken by him to bring an action for libel against Miss de Pledge in her capacity as Editor of the *Nurses' Journal*, the official organ of the association.

On being put the resolution was carried, out of sixty-nine medical men, matrons, and nurses present only one, Dr. Bedford Fenwick, voted against the motion.

The re-election of the honorary officers, as recommended by the Executive Committee, was next proposed. Dr. Bedford Fenwick objected on the ground that the honorary officers had not been nominated by the Executive Committee. It was then shown that the names of the whole of them were on the nomination paper, a copy of which was placed in the hands of each member of the Executive Committee when it last met.

H.R.H. Princess Christian (addressing Dr. Fenwick): Were you present at this meeting of the Executive Committee?

Dr. Bedford Fenwick: I was.

H.R.H. Princess Christian: As Dr. Fenwick was at the meeting of the Executive Committee and knew the honorary members had to be specially nominated, I wonder he did not say so, and not come here with a lawyer's letter in his pocket. (To Dr. Fenwick) Why did you not bring forward the matter at the meeting? (Cheers.)

A cordial vote of thanks was accorded to Sir James Crichton-Browne, the Princess in putting it to the meeting expressing the unabated confidence of the Council in him, and their grateful appreciation of the services he had rendered.

### BREAY v. BROWNE.

In the City of London Court on the 15th inst. Mr. Commissioner Kerr delivered his considered judgment in the case of "Breay v. Browne," a report of which appeared on p. 18 of the *Nursing Mirror* for the 10th inst. He said that he had considered how the verdict ought to be entered, and, after looking into the matter very fully, he must enter judgment for the plaintiff. There were two points. In the first place, there was rather a novel point as to what was the exact position of the subscribers to a chartered charitable incorporation like the Royal British Nurses' Association was, and the case itself was a matter of interest to a very considerable body—that was to say, of those who subscribed and became members of such a corporation and acquired certain rights. He would give leave to appeal, of course. He allowed costs on the higher scale, and granted a general stay of execution pending the appeal.

### The Poor Law Officers' Superannuation Act.

### MR. RUTHERGLEN AND "THE HOSPITAL."

We last week received a letter from Mr. John R. Rutherglen, in which he endeavoured to remove certain "misconceptions" as to the position of female Poor Law officers, and especially nurses under the "Poor Law Officers' Superannuation Act," and this week we have received a very indignant epistle in which he says: "I hope to receive an explanation of the omission of my letter last week, and an assurance that it will appear this."

Now the explanation is very simple, and explains its non-appearance this week also—the letter was far too long. We may add, however, that the fact that "the same communication" has been sent to "other papers" is only another reason why we should not find space for such widely-published matter.

In regard to the subject in hand, we quite admit that a nurse engaged under the Act may be held to have no "grievance" if she enters on the service with her eyes open, and if the guardians offer such an addition to the gross salary as "to make the net salary equal to what it was before the deduction under the Superannuation Act." We question, however, whether this will be done. Guardians are not apt to give more than the market price for any article, and if they only offer the outside price for nursing, either they must accept a poorer article or the nurses must have a grievance in regard to a "deduction" made on the plea of a pension which they will never receive.

One of the "misconceptions" which Mr. Rutherglen sets himself to refute is that the nurses "will be compelled to contribute out of their small pay towards a superannuation which few of them can hope to attain, and so to swell the pensions of the higher at the expense of the lower grades of the service." We cannot say he is very successful. We do not suggest that the pension of any clerk to the guardians will be increased by virtue of the "deductions" collected from all the scrubbers in the house; but we say in Mr. Rutherglen's own words, "The whole principle of the Act is based upon the theory that only a comparatively small proportion of those who contribute will ultimately need superannuation." . . . "Through death, resignation, or other causes, officers drop out before they arrive at the age or need of superannuation, and it is their payments that will provide a "certain" superannuation for their more fortunate brothers and sisters who outlive them and claim the benefits of the Act." If it had been merely a question of outliving them, not a word would have been said. What is said, however, is this: that the higher grades as a class do go on working up to a pensionable age, while the lower grades do not; and that the principle of the Act is the utilisation of the contributions of those who "drop out," viz., of the lower grades, to provide pensions for "their more fortunate brethren," viz., the higher grades, who stay on in the service and are thus able to claim superannuation. The superannuation of the upper grades is calculated upon this basis, and we thank Mr. Rutherglen for putting the matter so clearly. It now only remains for the guardians to accept his suggestion and replace in increased salary what is taken off in "deductions."

Until this is done we can but repeat our advice that nurses in the poor law service should avail themselves of their right to contract out, and that nurses who have not yet joined that service should not do so until the Act is altered.

### Where to Go.

Dowdeswell Gallery, 160, Bond Street.—At Dowdeswell's is now to be seen Herbert Schmalz's picture "Rabboni," the centre-piece of his two celebrated pictures, "The Return from Calvary" and "The Resurrection Morn," a work which completes the series painted by this artist, dealing with the latter events of Christ's life.

The Art and Crafts Exhibition at the New Gallery.—The Arts and Crafts Exhibition this year is full of interesting features. The exhibits are well displayed and not crowded together. Since the opening of the exhibition William Morris has passed away. On the glass cases, which contain specimens of his beautiful illustrated and typographical work, lies a wreath of bay leaves, encircling a touching tribute in verse, written by Walter Cane. The memory of another "arts and crafts" man is commemorated also by forming a special loan collection of his works. A large portion of his labours was devoted to designing cartoons for stained glass for Morris, and in these he shows some of his best work. There is something very beautiful in the simplicity and boldness of cartoon drawing, and admirers of this kind of work will find the new gallery very interesting this year. Not that by any means the display is limited to these. Beautiful copper, glass, wood, needle, iron, gold, glass and silver work are well represented. Amidst tapestry hangings, quaint furniture spinnets, and cases of pottery and bijouterie. Some of the workmanship in metals and jewellery especially demonstrate to us that neither art nor craftsmanship died when Italian art decayed. The "Arts and Crafts" are well worth a visit, if only that we may do justice in the future to the skilful hand and excellent brains of the craftsmen of our day. A quiet and delightful afternoon may be spent at the new gallery. What an excellent opportunity for change and recreation it affords to nurses, and to private nurses especially. It opens quite a fund of interesting topics wherewith to relieve the monotony of the sick room.

Royal British Nurses' Association.—We are requested by the Secretary to state that the first of the demonstrations on Invalid Cookery will be given at 17, Old Cavendish Street, on Tuesday, November 3rd, at half-past two. H.R.H. the President has fixed the date of the annual conversazione for Friday, December 18th. Members desirous of receiving their badges from the Princess on that occasion are requested to communicate with the Secretary.

### Appointments.

MATRONS.

Halstead Cottage Hospital, Essex.—Miss Alice Wakeling has been selected to fill the post of Matron at this hospital. She was trained at the Alexandra Hospital for Children with Hip Disease, London, and afterwards worked at the West Kent Hospital, Maidstone.

Joint Fever Hospital, Kilwinning.—Miss Mary B. Mitchell has been appointed to the Matronship of this new hospital. She was trained at the Kilmarnock Infirmary, where she has remained on the staff for the last six years, latterly in the capacity of ward sister. She goes to her new post with many wishes for future success.

Goole Cottage Hospital.—Miss Clara Hoadley has been appointed Nurse-Matron at this hospital. She was trained at Guy's Hospital, with which institution she has remained connected. Having served her five years, she holds a certificate and medal. Miss Hoadley carries with her to her new work many good wishes from her fellow-nurses.

Clapham Maternity Hospital.—Miss Wainwright has been appointed Matron of this hospital. She was trained at Pendlebury and at St. Bartholomew's Hospitals. She was for five years matron of the Jenny Lind Hospital, Norwich, and for the last two years has occupied the position of matron at St. John's House, Battersea, Maternity (a branch of the Clapham Maternity Institution).

Royal Hospital for Children and Women, Waterloo Bridge Road.—Miss Mary R. Easton, was elected Matron at this hospital on October 13th. Miss Easton is a Nightingale nurse. She was trained at St. Thomas's Hospital, where she was subsequently promoted to hold appointments as staff nurse and sister of wards. Miss Easton will be followed to her new work by many good wishes from her fellow nurses at St. Thomas's.

Rous Memorial Hospital, Newmarket.—Miss Augusta M. Hopkins has been appointed Matron of this hospital. She was appointed on October 15th. Miss Hopkins was trained at the Infirmary for Children, Liverpool. She was afterwards appointed on the extra staff of St. Thomas's Hospital, and worked as sister at Addenbrooke's Hospital, Cambridge, and at Victoria Hospital, Chelsea. She has also had some experience in district nursing.

### Novelties for Nurses.

HARTMANN'S PATENT WOOD WOOL PREPARATIONS.

It would be difficult to find anything more useful or efficacious for general purposes than these convenient and excellent preparations. Their great powers of absorption render them especially useful in surgical cases, while their antiseptic properties are of great value in the treatment of suppurating wounds, ulcerated surfaces, and cancerous growths. Free drainage is promoted, and all disagreeable smell removed by the use of these patents, of which there are several varieties, according to the nature and requirements of the case.

Wood wool tissue is a capital contrivance, whereby the wool is enclosed between layers of gauze. It can be cut into any shape as desired, and is very adaptable. It is sold in large sizes for accouchement purposes, and is the most comfortable and cleanly invention of the sort we have ever seen. The fact of its being perfectly antiseptic lessens the risk of puerperal and other complications. Furthermore, as it is completely absorbent, the necessity for the more costly macintosh is done away with, and the sheet can be burnt after use. This and other necessaries for the lying-in room n be procured in a neat case for one guinea, inclusive, thus saving much forethought and trouble in making the requisite preparations.

The Vaccination Pad is an ingenious contrivance, consisting of a square of the wool enclosed in gauze, and provided with tapes to secure it in position. It can be changed when dirty, thus reducing the risk of septic absorption to a minimum.

Sanitary Wood Wool Napkins (infant's napkins) are another development of this Protean production, and are deserving of special mention as effectual safeguards against the chafing and irritation so often produced by the necessarily constant changing of the linen articles in ordinary use.

Towelettes.—The advantage of these over the old-fashioned diaper are being constantly demonstrated. They are not only soft and very absorbent, but from a hygienic point of view are desirable in every way from their greater cleanliness and the ease with which they can be destroyed after use. We confidently recommend the various preparations of the Wood Wool Company to the attention of our readers, feeling sure that if they could be induced to give them a trial they would in no wise be disappointed. The depôt is at 26, Thavie's Inn, Holborn Circus, London, E.C.

### Minor Appointments.

Halstead Cottage Hospital.—Miss Isabel Sherlock has been appointed Nurse at this hospital. She received her training in Liverpool and Leicester, and afterwards held an appointment on the staff of the West Kent Hospital, Maidstone.

Herne Bay Cottage Hospital.—Miss Emma Cochrane has been appointed Nurse at this hospital. She received her training at the Belgrave Hospital for Children, and has also nursed at the Royal Infirmary, Windsor.

Poplar and Stepney Sick Asylum.—Miss H. C. Crick has been appointed Sister at this asylum. Miss Crick was trained at the Derby Royal Infirmary, and has also worked in this asylum as staff nurse.

Oldham Infirmary.—Miss Sophia Peters has been appointed Sister of of the women's and children's wards. Miss Peters was trained at this infirmary, after three years being promoted to the post of staff nurse, which she held for one and a half years, and earning during these four and a half years excellent testimonials from those under whom she has worked.

Blackburn Union Workhouse Infirmary.—Miss Annie Elizabeth Bartram has been appointed Superintendent Nurse at this Infirmary. She was trained at the West Derby Union Infirmary, and has since held appointments at the Mill Road Infirmary, Liverpool, as night charge nurse; as day charge nurse at the Toxteth Park Workhouse Infirmary; and at Hope Hospital, Salford Union; and as head nurse at the Newport (Mon.) Union Workhouse Infirmary.

Rotherham Hospital and Dispensary.—Miss Florence Brown has been appointed Night Sister at this hospital. After training at the Royal Albert Hospital, Devonport, for three years, Miss Brown was appointed head nurse at the General Infirmary, Northampton, where she remained two and a half years, subsequently taking up district work. Miss Brown has taken holiday duty at the Accident Hospital, Redruth, and holds a certificate from Queen Charlotte's Hospital and the L.O.S. diploma.

Thetford Nursing Association.—Sister Mabel Kennedy has been appointed to the post of District Nurse for Thetford She trained for two years at the York County Hospital, where she afterwards held, for three years, the posts of night-superintendent and ward sister; she also holds the certificate for maternity nursing from the General Lying-in Hospital, York Road, Lambeth, and first-class certificates for anatomy, physiology, nursing, hygiene, and massage. We wish Sister Mabel Kennedy every success in her new work.

### Everybody's Opinion.

### "THREE HUNDRED ORPHANS CAGED."

A Medical Correspondent writes: I regret to find that my communication (in your issue of the 3rd inst.) with reference to St. Mary's Home, Broadstairs, intended to point out the one blot noticed in its otherwise admirable arrangements, has stirred up rancorous feelings in the breasts of some who, like myself, have the real good of the institution at heart. I am obliged to the medical officer (Mr. Raven) for informing me—what was not pointed out at my visit—"that a large number of the cubicles open at once by a very simple contrivance," but am forced to ask why, if the contrivance for simultaneous opening be so simple it is not universally applied? "Nurse A. B." volunteers the statement that, of 300 cubicles, 30 have to be opened singly, and as these latter appear to contain some of the most helpless children I fear my anxiety as to the "risk of a holocaust of innocents"—for the meaning of which phrase the "shilling dictionary" may be serviceable—is not altogether unreasonable. Those of your subscribers who are curious in the matter can turn to the illustration which heads this letter, which is a sketch of similar cubicles at Kilburn, and can form their own opinions as to whether or not my comparing them to the "cages of a travelling menagerie" is justifiable. I do not waste your space in dealing with the panic and pneumonia theories of "Nurse A. B.," except to say that to provide against either risk "loving personal care" will prove far more efficacious than iron bars. I do not write without practical and extended experience in the responsible charge (by night as well as day) of children both helpless and mischievous; and I can unhesitatingly affirm that mere mechanical restraint is in such cases demoralising as well as dangerous. In conclusion, I would disclaim "animus" against an institution towards which I have myself contributed and caused others to contribute; and it is solely because I wish to avoid personalities that I again subscribe myself "A Medical Correspondent."

### THE POOR LAW OFFICERS' SUPERANNUATION ACT.

The Matron of the St. Marylebone Infirmary writes: The minds of those who have at heart the best interests of trained nurses in workhouse infirmaries are at present much occupied in considering the effect which the Poor Law Officers' Superannuation Bill is likely to have upon that very important branch of nursing work. The Act, as it stands, will benefit, with a very few exceptions, the "higher officials" only, the few exceptions being those nurses (or "officers," to use the technical term) who have now almost reached the age fixed for their retirement. All members of nursing staffs or servants who entered on duty before September 29th, 1896, may congratulate themselves that it is in their power to decline to become subject to the provisions of the Act. Three alterations appear to me to be essential before the Act can be considered otherwise than most unjust to all women workers under its provisions, be they nurses or servants. Firstly, that they should have the choice of availing themselves of it, or otherwise; secondly, that the age at which retirement becomes possible should be very considerably reduced; thirdly, that any nurse or servant desiring to withdraw from the poor law service should have the money which has been deducted from her salary returned to her. It is not to be supposed that the great body of trained nurses now in parish infirmaries all over England will be content to remain under such a condition of things; the injustice of the Act, however, to women workers is so palpable to all fair-minded people that it will, probably, before much time has elapsed, work its own remedy.

### Notes and Queries.

*Training.*

(28) Please tell me through your columns how I should set about gaining admission into the Children's Hospital, Broad Street, Birmingham, as a probationer. At what age are candidates eligible?—*A. W.*

Write to the matron for particulars. We do not know at what age probationers are taken at this hospital; but you will obtain all such information best direct from the matron.

*Monthly Nurses and Typhoid.*

(29) Please tell me if it is right for monthly nurses to take typhoid cases as intermediate work.—*Nurse M. J.*

No notice can be taken of letters which are unaccompanied by full name and address. See rules at head of this column.

*Incurable Home.*

(30) Can you tell me where a boy twelve years old, with hydrocephalus, could be taken for a small sum?—*A. G.*

You will find a list of suitable institutions in Burdett's "Hospitals and Charities" (Scientific Press, 28 & 29, Southampton Street, Strand, London, W.C.). Write to Miss Coleman, Hospital and Home for Incurable Children, 2, Maida Vale, London.

*Nursing Abroad.*

(31) Can you tell me how an English lady nurse can get work on the Continent, in a hospital or as nurse among English residents?—*S. H.*

We answer such queries as yours every week in these columns, and can only repeat that the number of nurses wishing to find work out of England is out of all proportion to the chances of so doing. You should watch the advertisements in The Hospital. There is certainly no demand for English nurses in continental hospitals, and unless you have an exceedingly sure prospect of work, nothing is more rash than starting private nursing on your own account "among English residents." In "How to Become a Nurse," and in "Hospitals and Charities," you will find the addresses of foreign institutions, and might apply to those where there are English matrons and nurses, but before taking any foreign appointment make most careful inquiries.

*Army Nursing Uniform.*

(32) Can you give me a full description of the uniform of an army nursing sister, the medals, and where these are placed? I wish to dress a doll for the *Truth* exhibition, and am very anxious to have it correct.—*Eva K.*

You will find a good many particulars about army nursing uniforms in "How to Become a Nurse" (Scientific Press, 28 & 29, Southampton Street, Strand, London, W.C.), in the chapter on "Army and Navy Nurses," and in that on "Uniforms." The latter gives illustrations of dress and medals. We think if you wrote to Miss Norman, Head of the Nursing Sisters, at Netley, Hants, explaining for what you require the information, she would be pleased to help you with further particulars.

*Training.*

(33) Will you tell me of some hospitals in London where non-paying probationers are received for general training? I am fairly well educated.—*S. W. H.*

Nearly all general hospitals in London take non-paying—or rather, paid—probationers for terms of three or four years' training. You had better read "How to Become a Nurse" (Scientific Press, 28 & 29, Southampton Street, Strand, London, W.C.). There you will find the particulars of the training given at the various hospitals, and should then write to the matrons for forms of application. We do not quite understand the rest of your letter, but if you mean that you have been told that domestic servants are not eligible as probationers, you are quite mistaken. At many hospitals matrons consider that women who have worked in that capacity make excellent nurses, provided they are, as you describe yourself, "fairly educated."

*Babies' Home.*

(34) I am a trained nurse, and I wish to take in young children to nurse. How should I make my wishes known, and how much ought I to charge for each child?—*Nurse L.*

If you start under public auspices you can advertise in The Hospital. But anything which trenches upon "baby farming" has become so unsavoury in the eyes of the public, that such an undertaking may rightly prove dangerous to the reputation if embarked on as a private venture.

## Figures and Tables

**Figure f1:**